# Unification of free energy minimization, spatiotemporal energy, and dimension reduction models of V1 organization: Postnatal learning on an antenatal scaffold

**DOI:** 10.3389/fncom.2022.869268

**Published:** 2022-10-14

**Authors:** James Joseph Wright, Paul David Bourke

**Affiliations:** ^1^Centre for Brain Research, University of Auckland, Auckland, New Zealand; ^2^Department of Psychological Medicine, School of Medicine, University of Auckland, Auckland, New Zealand; ^3^Faculty of Arts, Business, Law and Education, School of Social Sciences, University of Western Australia, Perth, WA, Australia

**Keywords:** free energy principle, spatiotemporal energy, dimension reduction, visual cortex, synchronous oscillation, apoptosis, cortical self-organization

## Abstract

Developmental selection of neurons and synapses so as to maximize pulse synchrony has recently been used to explain antenatal cortical development. Consequences of the same selection process—an application of the Free Energy Principle—are here followed into the postnatal phase in V1, and the implications for cognitive function are considered. Structured inputs transformed *via* lag relay in superficial patch connections lead to the generation of circumferential synaptic connectivity superimposed upon the antenatal, radial, “like-to-like” connectivity surrounding each singularity. The spatiotemporal energy and dimension reduction models of cortical feature preferences are accounted for and unified within the expanded model, and relationships of orientation preference (OP), space frequency preference (SFP), and temporal frequency preference (TFP) are resolved. The emergent anatomy provides a basis for “active inference” that includes interpolative modification of synapses so as to anticipate future inputs, as well as learn directly from present stimuli. Neurodynamic properties are those of heteroclinic networks with coupled spatial eigenmodes.

## Introduction

Explication of the stimulus filter characteristics of neurons has been a major theme in neuroscience for more than 50 years and studied in greatest detail in cortical area V1. This analysis has contributed significantly to the field of artificial neural networks, as well as visual processing. Yet puzzles in the organization of the filter characteristics have persisted, entwined with other puzzles—particularly the functional relevance of cortical columns and their variable definition in different cortical sites and species (Horton and Adams, 2005; Molnaìr, 2013)—leaving uncertain the ways real cortical mechanisms differ from the simplified solutions applied in deep learning backpropagation networks (Domingos, 2015; Marblestone et al., 2016). Selective filtering in real neurons has been carried over into artificial neural networks, but is this the only essential property? How much of the orderliness in mesoscopic cortical anatomy has functional importance? Is this order, or its lack, a co-incidental manifestation of growth processes and just a metabolically efficient arrangement, or is it essential to information processing *per se*?

Latterly, theoretical developments, based upon fundamentals of information processing, computation, and predictive coding, suggest that, *via* the Free Energy Principle and its concept of “active inference” (Friston, 2005, 2010; Clark, 2013), a deeper unification of brain structure and cognitive function may be possible. This abstract concept requires explication in cellular specifics—literally and metaphorically, flesh on its bones—but offers clarification of the goal to be achieved by models of the brain. Hopefully moving toward that goal, this paper extends our earlier “minimum free energy” (Wright and Bourke, 2021a,b) account of antenatal mesoscopic neocortical development into the postnatal period. We will show that, in this extension, further functional relations between the meso-anatomy of the cortex, the filter characteristics of cortical neurons, and of the storage and manipulation of sensory images become apparent.

### Feature filter models and problems encountered

From the foundational studies of Hubel and Wiesel, 1962, 1963, 1968, it was apparent that individual neurons responded to afferent pulses preferentially, as if filtering for selected characteristics, and were shown to exhibit anatomical order on the basis of these filter characteristics (Bonhoeffer and Grinvald, 1991 and subsequent)—whether OP, ocular dominance (OD), or the later emphasized SFP and TFP. Explaining how the selective characteristics developed was, and is, the central theoretical problem. A definitive review of early models, comparative and in historical order, is provided by Swindale (1996, 2008). A distinction may be drawn between models emphasizing feed-forward connections from the visual pathways, vs. those emphasizing contextual intracortical connections. The former class of models has been recently reviewed by Vidyasagar and Eysel (2015).

The initial feed-forward Hebbian models for OP share in common a conception of OP as consequent to the generation of a shaped field of excitation in small cortical areas and draw upon common assumptions of patterned retinal activity, Hebbian synapses, radially symmetric short-range excitatory and longer-range inhibitory lateral connections, and normalization of input strengths. Beginning from the work of von der Malsburg (1973), subsequent models in the family (von der Malsburg and Willshaw, 1976, 1977; Swindale, 1980, 1981a,b,^1982^; Linsker, 1986a,b,c; Miller et al., 1989; Obermayer et al., 1990, 1992; Goodhill, 1993) varied in learning rule details and updating, range of lateral correlation and inhibitory surround, nature of synaptic competition, distribution of synaptic terminals from afferents, correlation of binocular inputs, etc. All produced, to a varying degree, good accounts of the topology of OP, columnar order, and OD but did not easily explain why ordered OP emerged in the antenatal period without structured visual input (Wiesel and Hubel, 1974). Internally generated retinal waves were then supposed to provide the needed stimulus (Galli and Maffei, 1988; Burgi and Grzywacz, 1994). Yet the converse finding that visual stimuli are required to maintain the OP order in post-natal life (Hubel and Wiesel, 1970; Blakemore and Van Sluyters, 1974) even to the extent of requiring lines at particular orientations for the development of normal dendritic structure (Tieman and Hirsch, 1982) seemed contradictory if a simple stimulus were sufficient. A crucial assumption—that of a symmetric inhibitory surround extending beyond each zone of excitation—was not justified anatomically. Further, this class of models treated OP as a fixed filter property—not a property interactive with other stimulus contexts—and this was to prove problematic.

Another suggestion made early by Hubel and Wiesel was directed not to the origin of the filter properties, but their spatial ordering, and led to the development of another major idea (Kohonen, 1982; Mitchison and Durbin, 1986; Durbin and Willshaw, 1987; Durbin and Mitchison, 1990; Swindale et al., 2000)—that all combinations of different feature responses should be equally well represented over all positions in visual space. This would necessarily involve conflict at all points between continuity and completeness of all types of filtered representations—yet would favor minimization of axon and dendrite distances of connection between the cells. Conflict resolution required a packing of cells of different categories, constrained so as to fit all features closely together in the best approximation possible. This accounted well for the organization of OP about pinwheel singularities, linear zones, and saddle points, and could be seen to be operating to good effect at the margins of OD columns, and also at elevation/azimuth lines, in variants of V1 organization (Yu et al., 2005; Farley et al., 2007). It even accounted for extremes of either high space frequency preference (HFSP) or low space frequency preference (LSFP) about OP pinwheels (Issa et al., 2000), since this produces the best general matching of all OPs with all SFPs because of conflicts in attaining best continuity (Issa et al., 2008). As well as introducing a “small world” notion of cortical connections, the concept implied “dimension reduction,” since a higher dimensional feature space was being compressed onto the two-dimensional cortical surface. Associated ideas from information theory suggested that information coming from the retina is projected to the cortex with minimization of redundancy (Barlow, 1959) and conservation of maximum mutual information (Linsker, 1989). The dimension reduction model was compatible with feed-forward and Hebbian accounts but did not depend upon them, since it could be argued that the Hebbian group of models had been successful in the reproduction of OP and OD simply because they had each provided non-unique conditions imposing continuity and completeness on the outcomes. Similar issues are now emerging in machine learning in the form of disentangling representations in deep (convolutional) neuronal networks (Higgins et al., 2021).

Hebbian feedforward models then encountered another need for revision. The separate filter characteristics were interdependent, not independent. SFP, TFP, and stimulus velocity were interrelated because TFP was the optimum combination of stimulus space frequency and velocity (Baker, 1990). The OP preferences of neurons were not, as they had initially been assumed, fixed, simple responses to a single line. A neuron’s OP had been traditionally measured for slowly drifting stimulus lines oriented orthogonally to their direction of motion. However, OP varied systematically with speed of stimulus motion for all angles of attack other than that strictly orthogonal to motion, varying up to an OP orthogonal to that of the lowest speed (Basole et al., 2003, 2006). Prompted by this finding, the spatiotemporal energy model was advanced. This treated the individual neurons’ responses as a combination of their OP, SFP, and TFP responses, with spatiotemporal energy defined as the product of stimulus space frequency and speed. The individual cell’s responses could be predicted by summing feature preferences obtained from feature preference maps in the locale of the neuron (Zhang et al., 2007; Issa et al., 2008). When combined with the dimension reduction model, most problems seemed solved, but the origin of filter selectivity remained mysterious, and it was not entirely clear how OP and spatiotemporal energy were associated. An oddity not accounted for was that concurrent stimulation using stimuli with different orientation, yet all at optimum SFP, resulted in antagonistic blockade of responses, rather than independence or summation (Benevento et al., 1972; Blakemore and Tobin, 1972).

Arising from a rather different line of enquiry but motivated in part by the above problems, an account of the antenatal development of the neocortex was proposed by the present authors (Wright and Bourke, 2013, 2016, 2021a). The development of both columnar and of non-columnar cortex, the nature of superficial patch-to-patch connectivity, the organization of OP around singularities, OP linear zones and saddle points, like-to-like superficial patch/OP connections, and differences between monocular V1 and OD columns were explained. The model accounts for the emergence of ultra-small-world organization, and the generation of a transformed map of the visual input field—but does not depend upon structured input other than as diffuse noise. So, although consistent with continuity and completeness requirements, it is not a dimension reduction model in the usual sense. Synaptic competition and Hebbian learning are assumed, but the concept of an inhibitory surround is not required. The antenatal structure is considered a scaffold upon which postnatal organization can begin. The variation of OP with stimulus speed and angle of attack are explained, not as a consequence of combinations of features, but consequent to lag conduction within the superficial patch system. However, considerations of SFP and TFP were otherwise ignored. Consistent findings in non-columnar somatosensory cortex further supported the account (Wright et al., 2014) and it was later shown that the same principles can be extended from mesoscopic scale to inter-areal cortico-cortical connectivity (Wright and Bourke, 2021b). A link emerged to the very general, abstract, approach to learning proposed in the Free Energy Principle and related concepts of prediction error minimization and cortical computation, supplementing the earlier interpretations of continuity and completeness as redundancy minimization and maximization of mutual information.

### Summary of antenatal model

Our model is applied in the very sparse one-to-many connectivity of cortical neurons under unified fast and slow synaptic learning rules (Izhikevich and Desai, 2003) and neural dynamics,^1^ as summarized in Wright and Bourke (2021a,b). It has been observed that during embryogenesis synchronous firing of neurons protects them against apoptosis (Heck et al., 2008; Sang et al., 2021), as they form into small-world assemblies (Downes et al., 2012). This led us to propose that selection of developing neurons and synapses by apoptosis operates to maximize synchronous cell firing, thus shaping the outcome of genetically regulated cell numbers, patterns of cell migration, and differentiation into cell phenotypes (Rakic, 2009; Geschwind and Rakic, 2013). Synchronous oscillation is the “ground state” of equilibrium pulse exchanges among mixed excitatory and inhibitory cells (Chapman et al., 2002), so that, while constantly seeking equilibrium, the developing neurons also maximize their uptake of growth stimulation factors and thus tend to survive. Minimum resource consumption requires an approach to ultra-small-world configuration, further favoring avoidance of apoptosis early in embryogenesis. Extension of these principles would also regulate the generation and pruning of synapses at later stages.

In the developing cortex, early spontaneous synchrony comes under the influence of the sensory periphery as soon as afferents reach the cortex (Schmidt et al., 1999; Espinosa and Stryker, 2012; Molnaìr et al., 2020), and there is no clear transition from an antenatal to a postnatal state—merely an early phase and later stages through to adulthood. However, for purposes of convenience in the following account, we have referred to all later development once sensory inputs become structured as “postnatal,” although no definite time of transition between antenatal to postnatal is clear.

The early selection process is followed in a population of short and long-axon excitatory intracortical cells mixed with short-axon inhibitory partners. Polysynaptic flow in the one-to-many sparse connectivity of neurons leads to multi-stable equilibria of pulse exchange between all cells even though few are initially monosynaptically connected. This explains how long-range correlation of firing of developing neurons appears even before long-range connections are established (Smith et al., 2018).

Equilibrium requires the excitatory and inhibitory populations each fire in phase with cells of the same type, and in inverse phase between the two populations, so that early in development


(1) $$\begin{array}{c} \varphi_{i\,j}\,\left(t\right)=\varphi_{j\,i}\,\left(t\right) \end{array}$$

where φ_*ij*_ and φ_*ji*_ represent the exchanged pre-synaptic fluxes between *i-th* and *j-th* neurons over all pathways of connection. Competition and feedbacks inherent in synaptic learning rules lead toward bidirectional symmetry of gains along the prolific pathways, so a trend develops such that


(2) $$\begin{array}{c} \rho_{i\,j}\,g_{i\,j}\,\varepsilon_{i\,j}=\rho_{j\,i}\,g_{j\,i}\,\varepsilon_{j\,i} \end{array}$$

where ρ_*ij*,*ji*_ is the net structural synaptic connectivity between the two cells over all paths of connection, *g*_*ij,ji*_ is their slowly consolidated synaptic gain, and ε_*ij*,*ji*_ is fast transient synaptic efficacy. Each of the three factors converges on a separate time scale toward symmetry. Neurons unsuccessful in these competitive processes are eliminated, and initial, almost entirely unidirectional excitatory synaptic links become supplemented by an increased proportion of bidirectional monosynaptic connections, emerging from the polysynaptic background. Consistent with the Free Energy Principle, development follows a governing equation


(3) $$\begin{array}{c} F=A-C \end{array}$$

where *A* is the population sum of pulse autocorrelations, *C* is the sum of pulse cross-correlations, and *F*, the analog of thermodynamic free energy, is continuously minimized as bidirectional monosynaptic connections increase in number. This formulation of self-organization of the functional architecture of visual cortex reflects a key fact of the Free Energy Principle: many self-organizing systems move toward generalized synchrony and minimization of prediction errors, until all interactions have become established and reliable, and provide a complementary interpretation of Equation 3.

Freeenergy=accuracy⁢minus⁢complexity.

Accuracy (under the free energy principle) is the expected log likelihood of some observable outcome (e.g., presynaptic strengths), while complexity scores the divergence between posterior and prior representations of the latent causes of observable inputs. This can be read as the degrees of freedom that are induced by presynaptic inputs to cause a change in internal representations stored in a neuronal population.

**FIGURE 1:**
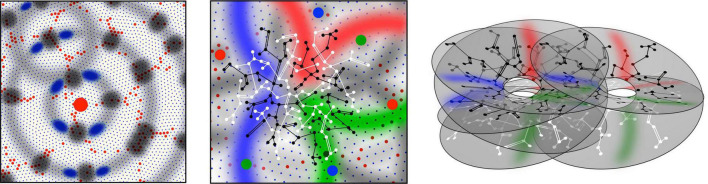
Patterns of synaptic connectivity seen in outcomes of growth simulations. **(Left)** Superficial patch cells. A representative long-axon (patch) cell (large red central spot) and patch connections. Surrounding zones of potential connection with other patch cells have been delineated in light gray concentric circles. Dark gray patches occur where other clusters of patch cells are positioned and able to make reciprocal connections, regularly spaced, patch-to-patch. Patch cell connections to short-axon local cells, maximizing resonance under stimulation of a particular angular domain are shown as darkened blue areas of “like to like” connections. **(Middle)** Local connectivity. Sparse short-axon cell connections have been marked in black or white, showing how interweaving networks occur. Some connections result in partial closure rather than complete independence of the interpenetrating networks. Fields of synaptic connections from patch cells to local cells are colored red, green, and blue according to their origins from diametrically opposite patch cell clusters These oppositely placed cell groups establish synapses on interpenetrating, distinct parts of the local cell network in a pattern best maximizing synchronous resonance, and creating local maps. **(Right)** A representation of intermingled networks of short-axon local cells conceptualized as cross-connected systems analogous to Mobius strips. Red, blue, and green bands indicate synaptic connections to/from the surrounding patch cell network. The degree of overlap of closed local cell connections can vary from clearly columnar to blurring with the apparent absence of columnar order.

The geometrical consequences for cell organization are indicated in Figure 1. Symmetry of the formation of synapses in small-world configuration requires the longer-axon cells to become superficial patch cells, forming patch-to-patch connections with other long-axon cells, while the short-axon cells form local clusters. Short and long-axon cells connect reciprocally at a range at which the population density of their axonal trees are similar, creating in the process an approach to classical “like to like” OP connections [although in this model, and in reality, patch cells communicate more broadly than strictly “like to like” (Martin et al., 2014)]. This results in the formation of “global to local maps,” where the “global map” is defined as the topography of an extended part of the cortical surface surrounding a local short-axon cluster, and the “local map” is the projection of the global map onto excitatory neurons of the local cluster. This leads to column formation, or to diffuse, apparently formless, connectivity, depending on the relative lengths of short and long axons—yet with the same pattern of small world organization—an order based on inverse synchrony-vs.-distance relations, synaptic competition, and local self-stabilization of pulse frequencies.

The emergent system provides lateral contextual information to neurons, determining their pattern of activation when they are also directly triggered by their extra-areal inputs. With regard to OP, coverage is both continuous and complete. This involves dimension reduction in a second sense, since, as bidirectional monosynaptic connections increase in number and free energy is minimized, system dimension falls.

The global to local maps are not simple Euclidean maps. Instead, they require projections to the inter-winding and cross-connected networks of local neurons and can be represented in the following mathematical form. *P* is a complex number position on the cortical surface and *p* is a complex number position within a local map with map center origin, *p_0*. The global map projection to any of many neighboring local maps takes the approximate form of projection of a Euclidean plane to intersecting Mobius strips, as


(4) $$\begin{array}{c} P\to \left\{p=\pm p^{{}^{\prime }}\frac{{\left(P-p_{0}\right)}^{n}}{{\left|P-p_{0}\right|}^{n-1}}+p_{0}\right\} \end{array}$$

where p′=-1k defines the rotation and scale of the local map, ± indicates map chirality, and *p*_0_ = *p*_0_ (1), *p*_0_ (2), *p*_0_ (3), are the local map centers. Symmetric reciprocal connections develop between superficial patch cells and local cells in arcs radiating from each map center, while maximum synchronous resonance requires the interpenetrating local networks are cross-linked into closed loops. Consequently *n* must take even integer values—the simplest case, *n* = 2, being that of projection to a single Mobius strip, or to multiple cross-linked Mobius strip-like networks. Other cases representing more complicated patterns of higher *n* may also be embedded and cross-linked with each other, but all appear similar to the simplest case, *n* = 2, when describing the appearance on the cortical surface, as if it were two dimensional.

Each inverse map, describing the return of reciprocal monosynaptic connections from local to patch cells, is given by


(5) $$\begin{array}{c} \mp P\leftarrow \pm \frac{1}{p^{{}^{\prime }}}\,{\left(\mp {\text{p-p}}_{0}\right)}^{\frac{1}{n}}\,{\left|\mp {\text{p-p}}_{0}\right|}^{n-1}-p_{0} \end{array}$$

The ∓ sign (distinct from the use of ± for map chirality) is here introduced because the input map results in coincident mappings (as viewed in two dimensions) to the 0 − π and π − 2π (i.e., + or −) “limbs” in the Mobius representation from the Euclidean global positions at angles 0 − 2π, thus creating the typical form of OP about a singularity at each map center.

Further maximizing synchronous resonance, adjacent local maps are arrayed in an approximately mirror-image formation, with cross-links between homologous map positions between neighbors. This results in the formation of linear zones and saddle points with, to greater or lesser degrees, interpenetration with other maps.

In proposing that developmental self-organization is based upon synchrony, we do not intend to exclude the possible relevance of alternative or complementary effects—as examples, organization of patch cell connectivity in a chemical diffusion model (Bauer et al., 2014), or recent revision of the retinal wave hypothesis (Kim et al., 2020)—and as a model of contextual interactions, there is some overlap with the model of Grabska-Barwinska and von der Malsburg (2008). However, the range of anatomical features explained by synchronous selection is so extensive that this model appears sufficient in itself.

### Requirements for approach to minimum prediction error

Overarching rules for adaptation included in the Free Energy Principle (Friston, 2005, 2010; Friston et al., 2012; Clark, 2013; Ramstead et al., 2018) help define goals for the outcome of the present model. The Free Energy Principle requires that, as learning progresses, the states of lower neural subsystems are precisely predicted, and their perturbing effects minimized, by subsystems higher in the sensory hierarchy. Zero prediction error requires that for any cortical area (with V1 representative) as external signals are input to the intracortical cells, signals later return from their distributions to the local maps to the sites of input in a precisely required match.

In the antenatal model, the bidirectional intra-areal exchange of signals can be represented as


(6) $$\begin{array}{c} O\,\left(P,t-\frac{\left|P-p\right|}{\nu }\right)\to \left\{o\,\left(p,t\right)\right\} \end{array}$$

and


(7) $$\begin{array}{c} \left\{o\,\left(p,t\right)\right\}\to O\,\left(P,t+\frac{\left|P-p\right|}{\nu }\right) \end{array}$$

where ν is the speed of intracortical signal conduction, {*o* (*p*, *t*)} are sets of synchronous pulse activity generated in the local maps, and $O\,\left(P,t\pm \frac{\left|P-p\right|}{\nu }\right)$ are the patterns of activity generating forward and backward pulse trains between sites of arrival of the input signals and the laterally distributed local maps. At an asymptotic limit of fully completed learning the difference in forward and backward signals must be minimized to zero in the face of ongoing perturbation by the inputs. That is


(8) $$\begin{array}{c} \left\{o\,\left(p,t\right)\right\}↔O\,\left(P,t\right)\,\forall \left(P,p\right) \end{array}$$

At that idealized limit, the input field and stored representations would exchange complete mutual information. This requires the exchanges must take place with group modes (eigenvectors of a delay matrix) that are invariant and bidirectionally symmetrical. There is an exact physical analogy to transmission without distortion of signals in fiber-optic cables, where absence of distortion (i.e., invariant group modes) requires continuous coupled interaction of spatial eigenmodes (Carpenter et al., 2016). So, our model may be expected to exhibit an analogous physiological expression of coupled spatial eigenmodes.

There are further demands to be made for a reasonably complete account. How will the newly induced selective-filter topography differ from the old? The stored information must enable association over both short and long ranges within the cortex. It should be seen how the antenatal organization provides a template for later development better than a random connectivity, and learning must converge more rapidly than a random walk.

## Postnatal development

### Spatiotemporal energy mapping *via* patch cells to local cells

We next consider the way in which inputs from the global field are conveyed to each local map.

Positions *P* (1) and *P* (2) on the cortical surface are crossed by a stimulus representation projected to the cortex, and convey pulses *via* superficial patch cells to a pair of closely situated local cells at positions *p* (1) and *p* (2) within any one of several local maps. We consider initially only the simplest cases, in which *p* (1) and *p* (2) pairs are always in the same limb of the same map. We need to determine conditions for arrival of synchronous, and near-synchronous, pulses at *p* (1) and *p* (2), since these will favor ongoing synaptic development within each local map.

**FIGURE 2:**
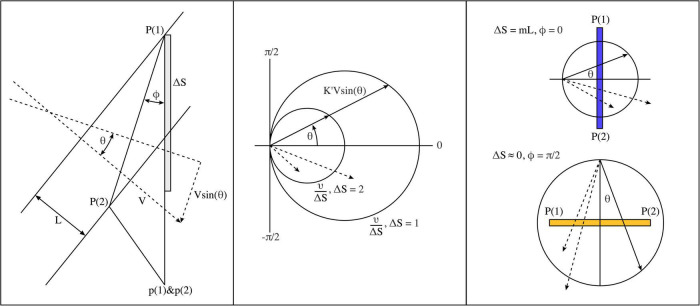
Geometric considerations determining the evolution of post-natal connectivity as structured external signals are imposed on the cortical field. **(Left)** Grating lines cross a pair of points, P (1) and P (2), in the global field, and axonal pulses are then relayed to closely situated local map cells, p (1) and p (2) *via* patch cell connectivity. **(Middle)** Polar diagram showing parameter combinations leading to synchronous pair arrival at p (1) and p (2). Circles show combinations resulting in synchronous pairs. Dashed vectors indicate a few of many possible asynchronous pulse arrivals at p (1) and p (2). **(Right)** Limiting cases of spatiotemporal orientation. Top: P (1) and P (2) are arranged radially to the local map singularity within which p (1) and p (2) lie. Bottom: P (1) and P (2) are arranged circumferentially.

Equivalent to a single space frequency in the representation of a moving object, consider a sinusoidal grating, with grating spacing *L*, and space frequency *K* = 1/*L*, moving over the surface at speed *V*, and angle θ to the line *P* (1) *P* (2) − itself oriented at an angle *ϕ* in the *P* plane relative to the local map in which *p* (1) and *p* (2) lie (Figure 2, left). For simplicity, assume one action potential pulse is generated each time a grating line crosses *P* (1) or *P* (2). Pulses will be generated at a rate *KV* at *P* (1) and *P* (2), and *KV* is spatiotemporal energy.

Inputs to *p* (1) and *p* (2) travel a distance Δ*S* further from *P* (1) than from *P* (2)


(9) $$\begin{array}{c} \Delta \,S=\left|P\,\left(1\right)-p\,\left(1\right)\right|-\left|P\,\left(2\right)-p\,\left(2\right)\right| \end{array}$$

so the difference in time of pulse travel to *p* (1) and *p* (2) from the respective source is


(10) $$\begin{array}{c} \Delta \,T=\frac{\Delta \,\text{S}}{\nu } \end{array}$$

where ν is the speed of axonal conduction.

As grating lines cross *P* (1) and *P* (2), each grating line will traverse along *P* (1) *P* (2) at a velocity *Vsin*θ, so the same grating line will generate pulses at *P* (1) and then *P* (2) after an interval δ*T*


(11) $$\begin{array}{c} \delta \,T=\frac{\left|P\,\left(1\right)-P\,\left(2\right)\right|}{V\,s\,i\,n\,\theta } \end{array}$$

|*P* (1) − *P* (2)| is necessarily some multiple of *L*, so


(12) $$\begin{array}{c} \delta \,T=\frac{m\,L}{V\,s\,i\,n\,\theta } \end{array}$$

Pairs of pulses must arrive at *p* (1) and *p* (2) with a time separation, λ


(13) $$\begin{array}{c} \lambda =\Delta \,T-\delta \,T=\frac{\Delta \,S}{\nu }-\frac{m\,L}{V\,s\,i\,n\,\theta } \end{array}$$

In the case that λ = 0, synchronous pulse-pairs arrive simultaneously at p (1) and p (2), and do so at a rate, ω, the rate of generation of synchronous pairs


(14) $$\begin{array}{c} \omega =\frac{1}{\Delta \,T}=\frac{\nu }{\Delta \,S}=\frac{K\,V\,s\,i\,n\,\theta }{m} \end{array}$$

The relative length, *m*, of *P* (1) *P* (2), has an effect equivalent to alteration of the spatial frequency, so writing *K*′ = *K*/*m*


(15) $$\begin{array}{c} \omega =\frac{\nu }{\Delta \,S}=K^{{}^{\prime }}\,V\,s\,i\,n\,\theta \end{array}$$

Since ω is a fixed function of Δ*S*, for any given *P* (1) and *P* (2), synchronous pairs can be created only for specific triplet combinations of {*K*, *V*, *sin*θ} (see Figure 2, middle). This means that as synchronous pair arrivals stimulate synchrony and encourage bidirectional synaptic connections among local neurons, they are also tuning these cells to specific combinations of spatiotemporal energy and the direction of object movement.

In all cases in which λ ≠ 0, pulse pairs reach *p* (1) and *p* (2) asynchronously, with either a lead or lag. Unidirectional monosynaptic connections will thus be promoted between *p* (1) and *p* (2), permitting the development of recurrent chains of connections promoting self-excitation among local cells. Self-exciting chains provide a basis for “winnerless competition” in synapse formation—an essential requirement of heteroclinic neural dynamics (Rabinovich et al., 2008). These considerations indicate that learned synaptic modifications will be capable of storing information about moving stimulus objects.

### Spatiotemporal orientation

It can be seen from Equations 13 and 14–15 that λ and ω vary continuously for small changes of Δ*S* and θ, and all four terms are dependent upon the alignment, *ϕ*, of *P* (1) *P* (2). Comparing the pairing of pulses generated from two closely situated pairs of cortical positions, *P* (1) *P* (2) vs. *P* (3) *P* (4), their difference in synchronous frequency is greatest when one pair of cortical positions is circumferential and one radially aligned (Figure 2, right). Conversely, afferent pulse pairs can approach concurrent synchrony when *P* (1) *P* (2) and *P* (3) *P* (4) are closely aligned and positioned. This relation of synchrony/asynchrony to alignment and position makes it helpful to define *ϕ* as the *Spatiotemporal Orientation* (STO).

Table 1 shows the bounds of the STO-related parameters, enabling these to be referred to as equivalents, according to the context.

**TABLE 1:** Bounds of STO.

ϕ (STO)	0	$\frac{\pi }{2}$
ΔS	mL	0
$\omega =\frac{\nu }{\Delta \,\text{S}}$	$\frac{\nu }{\text{mL}}$	∞

The effects of STO on the organization of connections among local neurons are discussed in the following section, “Concurrent evolution of local cell connections: Dimension reduction, minimized prediction error, and eigenmode dynamics,” and the implications for experimental findings in the sections subsequent, “Space frequency preference and temporal frequency preference experimental characteristics” and “Space frequency preference topographic order.”

### Concurrent evolution of local cell connections: Dimension reduction, minimized prediction error, and eigenmode dynamics

The impact of synchronous pair arrivals upon synaptic organization in the local map can be anticipated from the same neurodynamic principles applied in the antenatal model (Chapman et al., 2002; Wright and Bourke, 2021a). Cooperative processes of excitatory synaptic connection generation, and antagonistic excitatory/inhibitory interactions, must each be considered.

**FIGURE 3:**
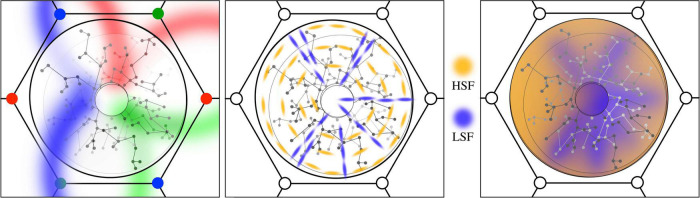
(Left) The antenatal local map. Like-to-like patch connections impose position and orientation on the global field onto the local map in radial form. (Middle) The early imposition of new patterns of local cell synaptic connection, based upon STO. Amber: local cells with circumferential orientation. Blue: radial orientation. (Right) Subsequent smoothing and dimension reduction *via* local synaptic connections. Zones of high and low spatial-frequency preference merge as local cell links provide smoothed interpolation between radial and circumferential extremes.

From the considerations in sections “Spatiotemporal energy mapping *via* patch cells to local cells” and “Spatiotemporal orientation,” the induction of synchrony among local cells by the arrival of synchronous pulse pairs must bring about synapse formation such that the spatial arrangement of *P* (1) and *P* (2), vs. *P* (3) and *P* (4) pairs becomes mapped onto the density of synaptic connectivity between corresponding *p* (1) and *p* (2) vs. *p* (3) and *p* (4) pairs—inducing a shift from the antenatal radial arrangement of “like to like” connections toward a revised system in which a new circumferential order is imposed upon the prior radial arrangement, as shown in Figure 3, left and middle. Thus, STO becomes an imposed local map property, as a continuous variable distributed over the complete antenatal small world representation of stimulus space.

As a secondary effect, some connectivity will also emerge between cells at *p* (1) and *p* (2) and those at *p* (3) and *p* (4) because of local interactions. The amplitude of synchrony between any two neurons reflects the “in-phase” (even) components received by each, with dissipation of odd components (Chapman et al., 2002) and in case of four cells, the degree to which all four achieve co-synchrony achieves the highest magnitude where the cells share a common resonance frequency—so locally generated synchrony between *p* (1) and *p* (2) and *p* (3) and *p* (4) bring about a partial merging of their STO responsivity, also weighted, in accord with their ultra-small-world organization, by their squared separation distance, *r*^2^. Certain *p* (1) and *p* (2) cell pairs that achieve early establishment of STO, {*ϕ*_*i*_}, by achieving the most stable pattern of co-synchrony over the local map, will force preliminary STO upon the more slowly developing connectivity. Provisional STO, {*ϕ*_*int*_}thus imposed, can be approximated by interpolation,^2^ as


(16) ϕi⁢n⁢t=a⁢r⁢g⁢∑i=1i=nϕi1+C⁢ri2

where *C* scales the range of interactions, but is without qualitative effects on the ordering of STO. The outcome in an example is shown in Figure 3, middle and right.

By this mechanism, local linkage between neurons of disparate STO will remain small compared to neurons of similar STO, achieving the compromise of continuity vs. completeness as well as smoothing and dimension reduction of the STO map. The modification and smoothing of STO at longer ranges has an important implication for learning because the property of spatiotemporal continuity at the level of the external stimulus world can be thus transferred to spatiotemporal continuity within the final STO organization of the local map—so as learning progresses, the interpolated circumferential/radial order must approximate more closely than chance the definitive order that will ultimately be attained. This constitutes a form of anticipatory prediction, minimizing future error, and facilitating Bayesian minimization during learning, not only minimizing prediction error on the basis of already-experienced inputs but anticipating aspects of the stimulus field not yet encountered.

As well as this cooperative organization of local connections, dynamic antagonism of radial and circumferential organizations must also arise, since these organizations share relatively few excitatory cross-links. Synchronous oscillation arises from equilibrium of exchange between both excitatory and inhibitory cells, with phase inversion between excitatory and inhibitory components (Wright and Bourke, 2021a), and it can be shown that where neurons lack strong excitatory cross-links, yet share interaction *via* intervening inhibitory short-axon cells, then equilibria can be reached by suppression of firing in either group by the other. Radially and circumferentially connected systems of neurons, engaging in crossed-inhibitory interactions, provide the anticipated analogy to coupled eigenmode dynamics, able to mediate the complicated time-sequences anticipated in heteroclinic dynamics. It may be noticed that this excitatory/inhibitory arrangement is not equivalent to the older concept of inhibitory surround.

### Space frequency preference and temporal frequency preference experimental characteristics

Comparisons can be made with experimental observations, where synaptic connections have formed as described above.

(i) A neuron driven from any global position *P* (1) by a drifting grid will respond by emitting pulses at frequency *KV* and will achieve maximum response at the frequency, ω, that best elicited synchronous resonance among the assembly of locally connected *p* (1) and *p* (2) pairs to which the stimulated neuron belongs—so exhibiting its TFP. That is, TFP = ω. TFP is more easily approached for cells with low TFP, given the relatively low stimulus speeds and wavenumbers that are experimentally practicable, compared to the high spatiotemporal energy required to approach TFP for cell with broad bandwidth, where Δ*S*→0. So, for HSFP cells, their TFP will generally be outside the experimental range.
(ii) *SFP* = *K* for given *V*, where *KV* = ω
(iii) Cells with broad bandwidth and thus high SFP will respond better to stimuli with a broad space frequency spectrum, and therefore more strongly to square waves than single sinusoidal inputs.

These properties can account for findings reported in Zhang et al. (2007) and Issa et al. (2008) in support of the spatiotemporal energy model. They explained response curves as composites of specific responses according to a combination of SFP and TFP. A strong TFP component (available by closer approach to ω in LSFP cells) explained the stronger recruitment of LSFP cells by increasing drift speed, compared to cells with HSFP, and the broader spatial bandwidth of square waves recruited HSFP neurons more than LSFP neurons.

Issa et al. (2008) also accounted for the change in OP with increasing stimulus speed and changes in stimulus angle of attack in spatiotemporal energy terms. As previously remarked, we have explained the same findings in terms of Doppler shift of lateral waves generated by a moving stimulus, without reference to OP/SFP linkage as such (Wright and Bourke, 2013). However, since both accounts successfully match the experiment, they can be considered equivalent time-series vs. Fourier explanations of the phenomena—or put in other terms, the Doppler-shifted spatiotemporal energy of input signals affects the SF and TFP of the local cells.

The crossed inhibition exerted each upon the other by circumferential and radially arrayed linked groups explains why response to concurrent presentation of stimulus grids of similar space frequency and speed, but differing orientations, produces not summation but cancelation of response—a property not otherwise explained in earlier models. This effect has further consequences for the topography of SFP.

### Space frequency preference topographic order

**FIGURE 4:**
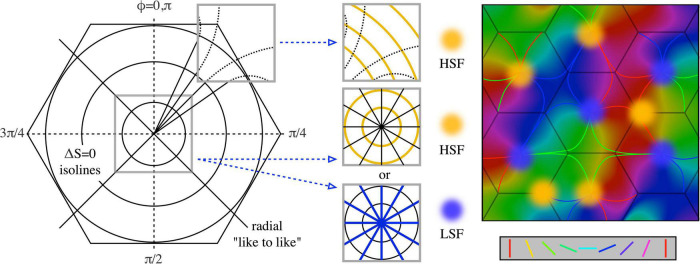
Synaptic competition and the emergence of experimentally observed spatial-frequency preference. (Left) Diagram shows the circumferential arrangement of p (1) and p (2) pairs responding to higher spatial frequencies, and the radial (along “like-to-like” lines) arrangement of pairs responding to lower spatial frequencies. At the periphery of the local map, like-to-like connections curve into a more circumferential array, as indicated by the dashed continuation of the radial lines. (Middle) Cut-out sections (top) show that on the local map periphery, where OP is normally continuous with that in the adjacent local map, circumferential pairs can be arranged contiguously with low conflict with radial arrangement. In contrast, near the singularity (lower cut-out sections) conflicts of radial and circumferential arrangement can lead to one or other of alternate HSFP or LSFP outcomes. (Right) Consequently, OP and high SFP are found together in OP linear zones, while SFP around the singularity must be either HSFP or LSFP (cp Issa et al., 2008).

Figure 4 shows the way in which SFP becomes topographically ordered in the way found experimentally and shows that the topological order reflects the degree of synergy or conflict between STO and OP responses in different situations. △*S* → 0 isolines can be constructed circling the singularity at all distances, and orthogonal to the antenatal radial like-to-like lines, for which △*S* ≈ *mL*. The antagonistic cross-inhibitory interactions of circumferential and radial arrangements induce conflicts near the singularity. For stable synaptic consolidations to be attained, distinct domains of either high, or of low, SFP, must appear randomly located around OP singularities, consequent to conflict resolution one way or the other. Conversely, the association of HSFP areas with OP linear zones also follows, as there is minimal conflict far from the singularity, where OP is itself essentially circumferential in relationship to the adjacent singularities. This can be seen in the form of the curved like-to-like connections shown in Figure 3 left, and the same effect is suggested by dashed curved lines in Figure 4, left and center. This joint alignment at the map periphery causes STO and OP to be synergic, both connection systems arising with low Δ*S*, and therefore SFP high.

With these extensions from the antenatal to the postnatal situation, the properties of SFP and TFP order are added to those of OP order. The present model thus incorporates the properties of both the dimension-reduction and spatiotemporal energy models.

### The storage of correlations at long range

Developing local connections permits association over short global distances, but how can learning of short-range correlations be generalized to association over the wider field of a cortical area? To answer this question, we consider the general case, in which cell pairs ∓*p* (1) and ∓*p* (2) are closely physically proximate in the cortical surface, but instead of being only close neighbors in the same limb of a single map as we first considered above—the two cells may be located in the same, or different limbs, within a single map, or in different intertwined maps, which may be of the same, or of different, chirality.

The way in which △*S* in the global field is related to distances within different local maps, or different limbs of the same local map, follows from the inverse maps (Equation 5)


(17) $$\begin{array}{c} △\,S=\left|\pm \frac{1}{p^{{}^{\prime }}}\,{\left(\mp p\,\left(1\right)-p_{0}\,\left(1\right)\right)}^{\frac{1}{n}}\,{\left|\mp p\,\left(1\right)-p_{0}\,\left(1\right)\right|}^{n-1}-p_{0}\,\left(1\right)\right|\\ -\left|\pm \frac{1}{p^{{}^{\prime }}}\,{\left(\mp p\,\left(2\right)-p_{0}\,\left(2\right)\right)}^{\frac{1}{n}}\,{\left|\mp p\,\left(2\right)-p_{0}\,\left(2\right)\right|}^{n-1}-p_{0}\,\left(2\right)\right| \end{array}$$

and similar considerations apply to the creation of synchronous pair inputs as in the simpler case.

Therefore the general case includes the possibility of long-range associations by local synaptic linkages—of disparate inputs from widely separated positions, differing relative orientations, and translations in the global field—all created by further cross-connections, breaking the antenatal Mobius-like order. The richness and range of cross connections that can be made in this way depend on overlap of local maps and suggest why the apparently random non-columnar order in most cortical areas may be functionally advantageous.

**FIGURE 5:**
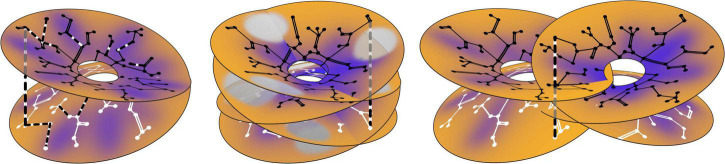
Establishment of long-range correlations. The antenatal neural connections are shown as in Figure 1 (right), and postnatal connection modifications are shown in dashed black and white. (Left) Within a single Mobius- like connection system, some antenatal connections are preferentially reinforced, while new post-natal connections also form, bridging the limbs of the earlier system. (Middle) Postnatal bridging connections establish further longer-range correlations within a multiplicity of such systems all surrounding a single singularity, each with only a partially complete representation of SFP. (Right) Further extending the possible range of association, overlapping local maps, surrounding separate singularities, are similarly brought into association by further postnatal bridges.

The positioning of cell positions with regard to breakdown of the Mobius order is shown in Figure 5.

## Conclusion

The goals for the extension of our antenatal model to postnatal development appear to have been met. Without the introduction of new assumptions, we have shown that the change from random noise inputs to structured inputs can transform the small world model of spatial positions and their short-range correlations to a finer grain of association at short and long ranges, and in temporal sequences. In this way, the antenatal structure acts as a scaffold able to guide finer resolution of spatiotemporal information within the pre-existing antenatal local maps.

This model conforms to expectation from the Free Energy Principle, with reduction of variational free energy and dimension reduction accompanying continually increasing mutual information between external inputs and the synaptic order, and provides a mechanism (coupled spatial eigenmodes) for asymptotic approach to zero “surprisal.” The previously unexplained observation that space-frequency-tuned responses delivered at multiple orientations block one another, seems to be of crucial significance, since this effect underlies the interaction of spatial eigenmodes.

It appears that the antenatal scaffold promotes later learning by “active inference” in ways that go beyond back-propagation in random networks, as usually conceived. First, the antenatal scaffold arising because of the declining synchrony-vs.-distance relationship general among cortical neurons establishes initial connections that conveniently approximate the topological order of generally declining cross-correlation-vs.-distance relationships of the sensory world in space and time. The initial antenatal order then gives way to postnatal connections that progressively represent ever more detailed partial correlations in the sensory world, superimposed upon, and given order by, the basic framework. Second, the establishment of later learning on the antenatal framework further exploits the cross-correlated structure of space and time to fill in tentative synaptic connections by the extrapolation mechanism described in section “Concurrent evolution of local cell connections: Dimension reduction, minimized prediction error, and eigenmode dynamics,” in advance of receipt of later inputs. That is, the general statistical order of the known is used to continuously update anticipation of the likely structure of the unknown. Generalizing to all subsequent exchanges within the cortical hierarchy, this would contribute a degree of flexible creativity to the brain’s self-supervision.

The long-standing interpretation of feature preferences as inherent filter properties of individual neurons is further qualified, and we have introduced a new concept, STO. Our account explains all the data incorporated in the spatiotemporal energy and dimension reduction models, and provides an explanatory mechanism for both, unifying this with other anatomical features explained by the earlier antenatal model. It does not purport to be an exhaustive model, of course. Discrepancies include the occurrence of OP fractures, and the occurrence of direction preference in some species, alluded to in Issa et al. (2008). Properties of the input pathways lying outside our consideration may account for these discrepancies.

Further testing of this model is within the realm of existing technologies. Further single cell testing using the methods described in Zhang et al. (2007) could test whether the postulated link between strength and preferred frequency of synchrony among cells in a small locale, and their individual TFP, is in fact the case. Detailed neurodynamic simulations are required to further demonstrate that the evolution of connections follows the paths we have here indicated. Synaptic architectonics at the micro- and meso-scales could be analyzed to see that the proposed general organization of local cells and patch cells follows the same form, whether the cortex is columnar or non-columnar, and accords with a Mobius-like organization. A relatively simple test would be to confirm or deny that superficial patch synapses from neurons on opposite sides of an OP singularity terminate on different “limbs” of the Mobius-like sheafs of local neuron connections.

As is emphasized in the Free Energy Principle, systems that learn—or develop—to minimize variational free energy are simply those in which members of an ensemble can predict each other accurately and with minimum complexity cost (i.e., maximum information and thermodynamic efficiency; Jarzynski, 1997). Since this phenomenon can be seen in *in vitro* cell cultures exposed to a structured input (Isomura and Friston, 2018) there is a possibility of testing the model by using structured inputs in cell culture preparations, to see what extent epigenetic scaffolding emerges and is necessary.

Although presented in terms of V1, there is reason to believe the model sufficiently general to apply throughout neocortex. Provisional extension to inter-areal interactions, and to computation mediated by inter-areal interactions (Perlovsky et al., 2011), as well as the necessary additional role of brain-stem mediated reward-based learning, have been discussed in association with other commentators in Wright and Bourke (2021b). If ultimately shown to be valid, this model may have implications for the further development of artificial intelligence, since it differs considerably from current orthodox deep learning networks, and, in its alliance with aspects of the Free Energy Principle, suggests the capacity for unsupervised learning.

## Data availability statement

The original contributions presented in the study are included in the article/supplementary material, further inquiries can be directed to the corresponding author.

## Author contributions

JW devised and wrote the manuscript. PB was responsible for software and graphics. Both authors contributed to the article and approved the submitted version.

## Footnotes

1 The neural field models upon which our arguments are based follow from the work of Freeman (1975) and Liljenstrom (1991), and their development is recounted in Wright (2016). They model the power spectrum, frequency wavenumber content, evoked responses, and synchrony of electrocortical waves (Wright and Liley, 1996; Robinson et al., 1997, 2001; Rennie et al., 2002; Chapman et al., 2002).

2 Values of *ϕ*_*i*_ were specified as unit vectors—either 1, 0 to represent a radial STO, or 0, 1, to represent a circumferential STO. These seeding values for interpolation were then used to compute interpolated STOs, {*ϕ*_*int*_}, throughout the local map, as arguments of weighted vector sums, in accord with Equation 16. This method utilizes the property that STO maps to positions in the local map, but is not a function of position—only of radial/circumferential orientation.
